# Genome-Wide Identification and Expression Analysis of the *CesA/Csl* Superfamily in *Madhuca pasquieri*

**DOI:** 10.3390/biology15120895

**Published:** 2026-06-06

**Authors:** Yule Chen, Jingzhe Qiu, Jiaxin Liu, Haoyou Lin, Lei Kan, Yihan Zheng, Jichen Wei, Lu Zhang

**Affiliations:** College of Forestry and Landscape Architectures, South China Agricultural University, Guangzhou 510642, China; llyychen@stu.scau.edu.cn (Y.C.); qiujingzhe@stu.scau.edu.cn (J.Q.); liujiaxin24@stu.scau.edu.cn (J.L.); llucky@stu.scau.edu.cn (H.L.); kanlei5523@163.com (L.K.); zhengyihan@stu.scau.edu.cn (Y.Z.); jichen@stu.scau.edu.cn (J.W.)

**Keywords:** *CesA*/*Csl* superfamily, cellulose synthase, glycosyltransferase, cell wall, secondary wall, phylogenetic analysis, *Madhuca pasquieri*, plant growth

## Abstract

The cellulose synthase gene superfamily encompasses two major groups, *CesA* and *Csl*, which are vital for synthesizing cellulose and hemicellulose in plant cell walls and fundamental to plant growth and developmental regulation. *Madhuca pasquieri* is a rare tree with high timber value. Currently, there is no relevant report on the identification and characterization of the *CesA*/*Csl* gene family in *M. pasquieri*. In this study, based on the high-quality genome of *M. pasquieri*, 47 members of the *CesA*/*Csl* superfamily were identified and classified into seven subfamilies, including *CesA*, *CslA*, *CslB*, *CslC*, *CslD*, *CslE* and *CslG*. Cis-acting elements were identified via analysis of the 2000 bp upstream sequences of *MpCesA*, suggesting extensive involvement in biotic and abiotic stress regulation. Based on the transcriptome data of five growth periods, the expression of the *CesA*/*Csl* family was analyzed. Combined with phylogenetic information, it is inferred that *MpCesA4*/*7b*/*7a*/*8b* may regulate the secondary wall, while *MpCesA1*/*3b*/*6b* may regulate the primary wall. Protein–protein interaction showed that *MpCesA4*/*7b*/*8a* were in the core site. Finally, we constructed the cellulose synthase complex (*MpCesA4*/*7b*/*8b*) model using AlphaFold3, which suggests that *MpCesA4*/*7b*/*8b* may form a complex on the plasma membrane to carry out cellulose synthesis. This study has a limitation in that the complex and its expression lack experimental validation, and only data analysis is provided as a reference, offering some directions for future research. In summary, the systematic characterization of the *MpCesA*/*Csl* gene family provides important insights into cell wall formation, genetic enhancement, and future biotechnological applications of this species.

## Simple Summary

Cellulose synthase genes are essential for plant cell wall formation and plant growth. For the rare and valuable timber tree *Madhuca pasquieri*, the *CesA*/*Csl* gene family has not been studied before. In this research, a total of 47 *CesA*/*Csl* genes were identified in *M. pasquieri* and divided into seven subfamilies. Cis-acting elements suggested extensive involvement in biotic and abiotic stress regulation. Transcriptome analysis across five growth stages revealed different expression patterns of these genes in primary and secondary cell wall regulation. Several core genes were screened out via phylogenetic analysis and protein interaction analysis. Furthermore, a cellulose synthase complex model was built, suggesting that specific proteins may form complexes on the cell membrane to synthesize cellulose. This study systematically analyzes the *CesA*/*Csl* gene family in *M. pasquieri*, offering valuable references for its cell wall research and genetic improvement.

## 1. Introduction

The plant cell wall determines plant morphogenesis and plays an essential role in plant growth and development [1]. Its main components consist of cellulose, hemicellulose, pectin [2], and lignin [3]. Plant cell walls, classified as primary and secondary, respectively, enclose growing and differentiating cells and provide structural support for xylem and plant tissues [4,5]. The secondary cell wall is deposited between the plasma membrane and the primary cell wall after cell expansion ceases, and is predominantly composed of cellulose [6]. As one of the most abundant renewable resources on Earth, plant cellulose provides valuable raw materials for industry, textile production and other fields [4]. Accordingly, research on the plant cell wall and its biosynthesis has become a core research hotspot in plant science.

The *CesA*/*Csl* superfamily belongs to the Glycosyltransferase 2 (GT2) family. It could be divided into the *CesA* and *Csl* subfamilies. It is widely acknowledged that *CesA* genes encode enzymes responsible for cellulose synthesis, while *Csl* genes mediate the biosynthesis of hemicellulosic polysaccharides. With the progress of research, numerous *CesA*/*Csl* genes have been successively identified in a wide range of plant species. They were initially reported in plants such as *Gossypium hirsutum* [7,8], *Arabidopsis thaliana* [9], *Oryza sativa* [10], and *Populus trichocarpa* [11]. These genes have now also been characterized in *Medicago sativa* [12], *Nicotiana tabacum* [13], *Solanum Lycopersicum* [14], *Vitis vinifera* [15], and *Eucalyptus grandis* [16].

*CesA* genes have been extensively characterized in higher plants [17]. *A. thaliana* contains 10 *CesA* members, whereas poplar harbors 17 *CesA* genes [18]. In *A. thaliana*, *AtCesA1*, *AtCesA3* and *AtCesA6* are indispensable for primary cell wall formation, while *AtCesA4*, *AtCesA7* and *AtCesA8* are critical for secondary cell wall biosynthesis [19,20,21,22]. *CesA2*, *CesA5* and *CesA9* exhibit partial functional redundancy with *CesA6* [23]. In addition, the transcription of *AtCesA4*/*7*/*8* is regulated by the AtMYB46 transcription factor [24].

Before catalyzing cellulose synthesis, cellulose synthases must first assemble into the cellulose synthase complex (CSC) [25]. CSC is plasma membrane-localized, dynamic high-order oligomers with hexagonal symmetry, presenting a rosette-like structure [26]. This rosette architecture was first observed on the plasma membrane of *Zea mays* and *Pinus taeda* using freeze-fracture electron microscopy [27]. To date, the plant CSC has been confirmed as a hexameric rosette structure, although the exact number of *CesA* subunits remains controversial.

The *Csl* family comprises nine subfamilies: *CslA*, *CslB*, *CslC*, *CslD*, *CslE*, *CslF*, *CslG*, *CslH* and *CslJ* [28,29]. Studies have shown that the *CslA* gene participates in the biosynthesis of mannose and glucomannan backbones [30,31]. The *CslC* gene is responsible for the biosynthesis of the β-1,4-glucan backbone required for xyloglucan synthesis [32]. The *CslD* gene may be involved in the synthesis of cellulose or mannose and functions in tip-growing cells [33]. In contrast, the *CslF*, *CslH* and *CslJ* subfamilies catalyze the formation of (1,3;1,4)-β-glucan [34,35]. The specific functions of the other four *Csl* subfamily members, including *CslB*, *CslE*, *CslG* and *CslM*, remain unclear. In *A. thaliana*, the *Csl* family contains 30 members categorized into six subfamilies: *CslA*, *CslB*, *CslC*, *CslD*, *CslE* and *CslG* [36].

As a member of the Sapotaceae family, *Madhuca pasquieri* is a rare tree species with important ecological value. Classified as Vulnerable (VU) on the IUCN Red List, it is also designated as a national Class II key protected wild plant and a species with extremely small populations in China and was added to China’s National Protected Forest List in 2025. This species naturally occurs in Guangdong, Guangxi and Yunnan of China, as well as northern Vietnam. It also boasts high economic value: its seeds have an oil content of around 30%. Its dense, hard and wear-resistant wood makes it a prized timber, widely used to produce furniture, equipment and veneers. At present, *M. pasquieri* faces multiple threats, including slow seedling growth, continuous population decline, and severe conflicts between conservation and rational utilization. Existing studies mainly focus on its morphological characteristics, artificial cultivation, transcriptomics and metabolomics analysis [37,38]. However, in-depth research on the molecular mechanism underlying its growth remains insufficient.

With the advent of next-generation sequencing technology, the availability of high-quality genome assembly for *M. pasquieri* has laid a solid foundation for in-depth research on gene families. However, the *MpCesA* gene family in *M. pasquieri* has not yet been comprehensively identified and characterized. Using the *M. pasquieri* genome as a reference, we performed a genome-wide analysis of the *MpCesA* gene family via bioinformatic tools. The objectives of this study were as follows: (1) phylogenetic analysis, chromosomal localization and collinearity analysis of *MpCesA* genes in *M. pasquieri*; (2) gene structure characterization and prediction of protein tertiary structures; (3) transcriptional expression profiles across different growth stages. The absence of qRT-PCR validation is a limitation of this study.

## 2. Materials and Methods

### 2.1. Identification and Physicochemical Prediction of MpCesA/Csl Superfamily

The genome of *M. pasquieri* used in this study has been deposited in the Genome Warehouse of the National Genomics Data Center (https://ngdc.cncb.ac.cn), China National Center for Bioinformation. Its corresponding accession number is GWHHIXU00000000.1. *CesA*/*Csl* protein sequences of *A. thaliana* were retrieved from the NCBI database (https://static.pubmed.gov/portal/portal.fcgi/, accessed on 14 April 2026) (Table S1). BLAST screening was conducted on the longest protein isoforms of all *M. pasquieri* genes with *AtCesA*/*Csl* sequences as queries using TBtools-II software (v2.467) [39]. The E-value threshold was set to 1 × 10^−5^ to screen candidate *MpCesA*/*Csl* members. Conserved domains of all candidate sequences were examined via the NCBI Conserved Domain Database (https://www.ncbi.nlm.nih.gov/Structure/cdd/, accessed on 14 April 2026), and sequences without complete conserved domains were eliminated to determine the final *MpCesA*/*Csl* family members. Physicochemical characteristics of *MpCesA*/*Csl* proteins were predicted using the ExPASy online server (https://web.expasy.org/protparam/, accessed on 14 April 2026) [40]. Subcellular localization patterns were analyzed through the WoLF PSORT online platform (https://wolfpsort.hgc.jp/, accessed on 14 April 2026) [41].

### 2.2. The Phylogenetic Tree Construction of the MpCesA/Csl Family

To explore the phylogenetic relationships of the *CesA*/*Csl* gene family, *CesA*/*Csl* amino acid sequences from *A. thaliana*, *P. trichocarpa* and *V. vinifera* were collected for phylogenetic tree construction (Table S1). Sequence alignment and similarity analysis were conducted using MEGA 11. The phylogenetic tree was constructed in FastTree 2.2 with the maximum likelihood method and 1000 bootstrap replicates, and all remaining parameters kept default settings [42]. The resulting phylogenetic tree was further visualized and optimized on the Chiplot online platform (https://www.chiplot.online/) [43].

### 2.3. Gene Structure, Conserved Motif and Conserved Structural Domains Analysis

Conserved motifs of all *MpCesA*/*Csl* family members were predicted using the MEME online tool (http://meme-suite.org, accessed on 14 April 2026) [44]. The number of predicted motifs was set to 10, and all other parameters were kept at default values. The gene structure information and conserved motif data of *MpCesA*/*Csl* were integrated for subsequent analysis. Combined results of gene structure and conserved motifs of *M. pasquieri* gene family were visualized with TBtools-II software.

### 2.4. Chromosomal Distribution and Synteny Analysis of MpCesA/Csl Family

Chromosomal localization of 47 *MpCesA*/*Csl* genes was performed in TBtools-II based on genomic annotation information. In the “gene density” module of TBtools-II, gene density was calculated with the bin size set to 100,000, and all other parameters remained as default. The gene density files were further applied to optimize the chromosomal localization map. Gene sequences and annotation files of *M. pasquieri*, *A. thaliana* and *P. trichocarpa* were imported into the “One Step MCScanX” module of TBtools-II for collinearity analysis. The interspecific collinearity relationships were visualized by the Dual Synteny Plot. Intraspecific collinearity within the *M. pasquieri* genome was analyzed via “One Step MCScanX”, and intraspecific synteny was displayed using “Advanced Circos”. During the analysis, the BlastP CPU value was set to 2, and the E-value threshold was set to 1 × 10^−10^.

### 2.5. Cis-Acting Element Analysis of MpCesA/Csl Family

The 2000 bp upstream sequences of each *MpCesA*/*Csl* gene were extracted from the *M. pasquieri* genome as promoter regions. The online tool PlantCARE (https://bioinformatics.psb.ugent.be/webtools/plantcare/html/, accessed on 16 April 2026) was used to predict cis-acting elements in these promoter regions [45]. The results were visualized using TBtools-II software.

### 2.6. Expression Analysis of MpCesA/Csl Family

Transcriptome sequencing data were obtained from our previously published research [38], specifically as follows. *M. pasquieri* was cultivated in an artificial climate chamber at South China Agricultural University. The controlled growth conditions included a constant temperature of 25 °C, relative humidity ranging from 60% to 80%, a 14 h light and 10 h dark photoperiod, and a light intensity of 17,600 lx. Uniform seedlings cultivated with the same substrate were collected at five key developmental stages after germination, namely seed germination, hypocotyl elongation, epicotyl elongation, two-leaf stage and nine-leaf stage. Three independent biological replicates were prepared for each stage. All whole-plant samples were immediately frozen in liquid nitrogen and preserved at −80 °C for subsequent experiments. In the present study, we extracted the FPKM expression values of the *MpCesA*/*Csl* gene family (Table S2). All expression data were standardized with the calculation of log2(expression value + 1), and the resulting data were visualized using the online Chiplot platform (https://www.chiplot.online/index.html, accessed on 20 April 2026).

### 2.7. Protein–Protein Interaction Network of the MpCesA/Csl Family

To construct the protein–protein interaction (PPI) network of *MpCesA*/*Csl* family proteins, all *MpCesA*/*Csl* protein sequences were submitted to the STRING 12.0 database (https://string-db.org/) [46]. Homology mapping was performed using *A. thaliana* as the reference organism, with the interaction confidence score set to 0.4 and other parameters kept as default. The resulting interaction data were downloaded and imported into Cytoscape 3.9.1 software for visualization and esthetic refinement [47].

### 2.8. 3D Structure Analysis of Proteins of the MpCesA/Csl Family

Representative protein structural models were predicted using AlphaFold3 (https://alphafoldserver.com) [48]. AlphaFold3 was also applied to predict the structural conformation of protein complexes formed by *MpCesA4*, *MpCesA7b* and *MpCesA8b*. All predicted structural results were refined and visualized with PyMOL 3.0 software.

## 3. Results

### 3.1. Identification of CesA/Csl Genes and Property Prediction in M. pasquieri

In this study, a total of 47 *CesA*/*Csl* proteins were successfully identified in *M. pasquieri* through sequence alignment and manual domain verification. Following the nomenclature system for this gene family in the model plant *A. thaliana*, we assigned corresponding names to these genes in *M. pasquieri*. Among them, 14 *CesA* genes were designated as *MpCesA1a*, *MpCesA1b*, *MpCesA3a*, *MpCesA3b*, *MpCesA4*, *MpCesA6a*–*6e*, *MpCesA7a*, *MpCesA7b*, *MpCesA8a*, and *MpCesA8b*. The other 33 genes belonged to the *Csl* subfamily and were named *MpCslA1*–7, *MpCslB1*–2, *MpCslC1*–7, *MpCslD1*–8, *MpCslE1*–4, and *MpCslG1*–5 (Table 1). Analysis of the physicochemical properties of the *MpCesA*/*Csl* proteins revealed that the number of amino acids ranged from 404 (*MpCslA7*) to 1173 (*MpCslD7*). Isoelectric point (pI) analysis showed that 15 genes had a pI below 7, while 32 genes had a pI above 7, indicating that the *MpCesA*/*Csl* gene family mainly consists of basic proteins. The aliphatic index varied from 76.61 (*MpCslD6*) to 105.18 (*MpCslA6*), reflecting substantial differences in the thermal stability of proteins within this family. In terms of hydrophilicity analysis, 30 *MpCesA*/*Csl* genes exhibited negative values, indicating their hydrophilic nature (Table 1), with approximately two-thirds of the *CesA*/*Csl* proteins being hydrophilic. Subcellular localization prediction indicated that these proteins are most likely located on the plasma membrane.

### 3.2. Phylogenetic Analysis and Classification of MpCesA/Csl Family

**Figure 1:**
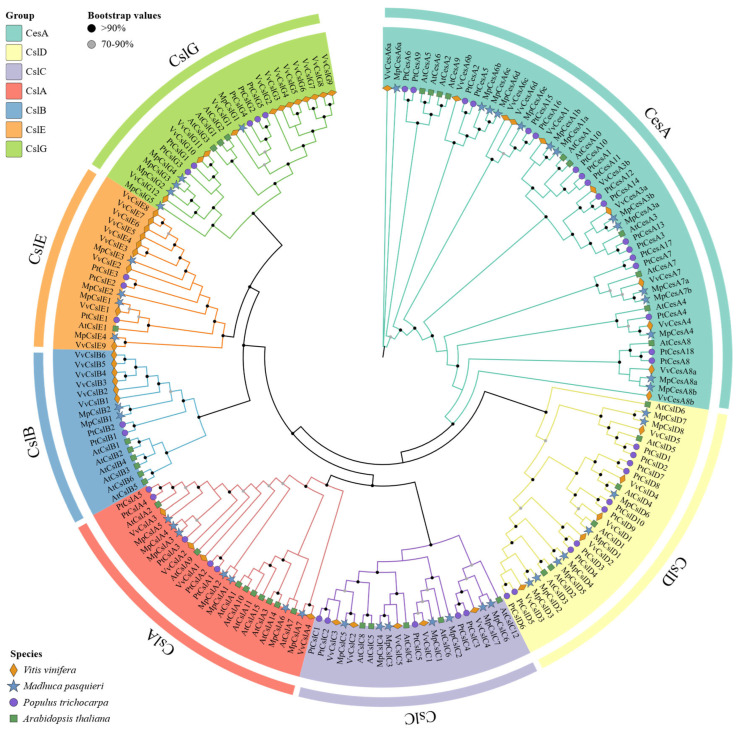
A phylogenetic tree was constructed for *CesA*/*Csl* genes from *M. pasquieri*, *A. thaliana*, *P. trichocarpa*, and *V. vinifera*. Genes from different species are represented by distinct symbols: blue pentagrams for *M. pasquieri*, green squares for *A. thaliana*, purple circles for *P. trichocarpa*, and orange diamonds for *V. vinifera*.

To investigate the functions of *MpCesA*/*Csl* proteins, a phylogenetic tree was constructed using TBtools-II based on 187 protein sequences from *M. pasquieri*, *A. thaliana*, *P. trichocarpa*, and *V. vinifera* (Figure 1). The results showed that all *CesA*/*Csl* proteins could be divided into seven subfamilies, including one *CesA* subfamily and six *Csl* subfamilies (*CslA*, *CslB*, *CslC*, *CslD*, *CslE*, and *CslG*). The *MpCesA* subfamily contained 14 members, while the *MpCslA* subfamily had seven members, the *MpCslB* had two, *MpCslC* had seven, *MpCslD* had eight, *MpCslE* had four, and *MpCslG* had five.

### 3.3. Analysis of Gene Structure and Conserved Domains of the MpCesA/Csl Family

**Figure 2:**
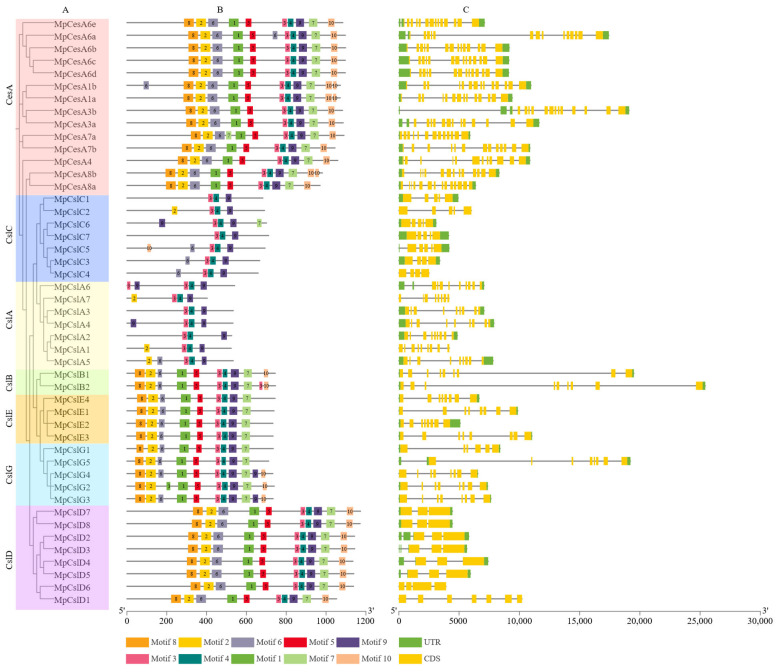
The phylogenetic tree, conserved amino acid motifs and gene structure of *MpCesA*/*Csl* proteins. (**A**) Phylogenetic tree of *MpCesA*/*Csl* proteins, where seven different colors represent seven subfamilies. (**B**) Conserved amino acid motifs of *MpCesA*/*Csl* proteins, with ten different color blocks representing different motifs. (**C**) Gene structure of *MpCesA*/*Csl* proteins, in which the yellow regions represent coding sequences (CDS) and the green regions represent untranslated regions (UTR).

Forty-seven *MpCesA*/*Csl* proteins were divided into seven categories in the phylogenetic tree (Figure 2A). The conserved motifs of these 47 *MpCesA*/*Csl* proteins were identified using the online MEME website (Figure 2B). We set the motif identification parameter to 10, and a total of 10 conserved motifs were identified accordingly. Conserved motif analysis showed that almost all *CesA*/*Csl* proteins contain motif 3, motif 4, and motif 9, suggesting their conservation and functional similarity as members of the same family. Most proteins in the *CesA*, *CslB*, *CslE*, *CslG*, and *CslD* subfamilies contain motif 8, motif 2, motif 6, and motif 1. In contrast, the *CslA* and *CslC* subfamilies do not have these motifs; the difference in conserved motifs may indicate functional divergence among their subfamilies. In addition, the exon–intron distribution pattern of *MpCesA*/*Csl* genes was investigated. As shown in Figure 2C, we found that the number of exons varies among different subfamilies. For example, most members of the *MpCslD* subfamily have only three exons, while most members of *MpCesA* have more than ten exons.

### 3.4. Chromosomal Localization and Collinearity Analysis of the MpCesA/Csl Family

**Figure 3:**
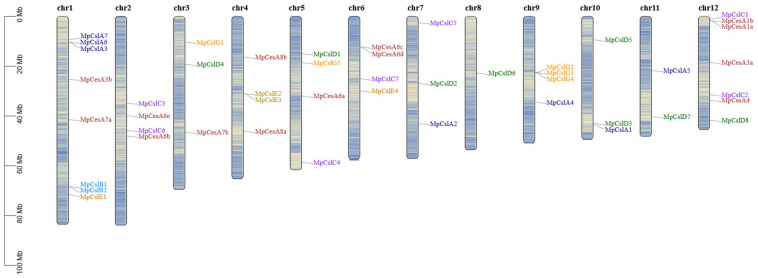
Chromosomal locations of *MpCesA*/*Csl* genes. Chromosome numbers are displayed above each chromosome. The scale bar represents the length in megabases (Mb). The red regions on each chromosome indicate high gene density, while the blue regions indicate low gene density.

According to annotations, 47 genes are distributed across 12 chromosomes (Figure 3). The number of *CesA*/*Csl* genes on each chromosome ranges from 1 to 8. Among them, chromosome 1 contains the largest number (eight genes), while chromosome 8 contains the smallest number (one gene). These 47 genes exhibit diverse distribution patterns, including clustered and isolated distributions. Among them, *MpCslA3*/*MpCslA6*, *MpCslE2*/*MpCslE3*, *MpCesA6c*/*MpCesA6d*, *MpCslG3*/*MpCslG4*, and *MpCesA1a*/*MpCesA1b* are described as tandem duplicate gene pairs.

**Figure 4:**
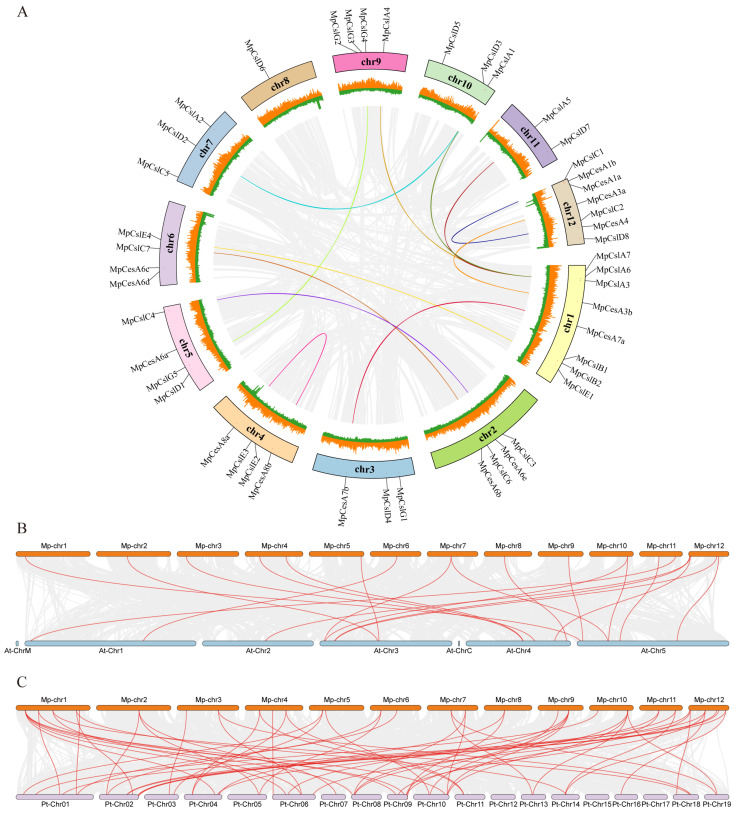
Intraspecific and interspecific collinearity analysis of *M. pasquieri*. (**A**) Intraspecific collinearity analysis. Colored lines indicate segmental duplication events of the *MpCesA*/*Csl* gene family, and gray lines represent genome-wide collinear blocks. The outer orange peaks show gene density, and the inner green peaks represent GC content distribution. (**B**) Interspecific collinearity between *M. pasquieri* and *A. thaliana*. Gray lines indicate genomic collinear blocks, and red lines mark the homologous genes of the *MpCesA*/*Csl* family. (**C**) Interspecific collinearity between *M. pasquieri* and *P. trichocarpa*. The meanings of gray and red lines are consistent with the above.

In addition to five tandem duplication pairs, we also investigated segmental duplication events within the *MpCesA*/*Csl* gene family (Figure 4A). Intraspecific collinearity analysis identified 12 segmentally duplicated gene pairs in this gene family. Comprehensive analysis revealed that segmental duplication acts as the primary driving force for the expansion of the *MpCesA*/*Csl* gene family. Furthermore, we calculated the Ka/Ks values of tandem and segmental duplicated gene pairs to determine the selective pressure underlying duplication events (Table 2). All gene pairs presented Ka/Ks ratios lower than 1, indicating that these genes have experienced purifying selection and possess functionally conserved characteristics.

To further clarify the phylogenetic relationships of the *MpCesA*/*Csl* gene family, comparative collinearity maps were constructed by integrating *M. pasquieri* with two representative species, *A. thaliana* and *P. trichocarpa* (Figure 4B,C). The results identified 20 pairs of *CesA*/*Csl* homologous genes between *M. pasquieri* and *A. thaliana*. In addition, a total of 59 collinear gene pairs were detected between *M. pasquieri* and *P. trichocarpa*. These findings indicated that the *CesA*/*Csl* gene families of the two species have undergone frequent chromosomal fragment rearrangement, gene duplication, and gene loss during evolution. Accordingly, it is speculated that *P. trichocarpa* shares a closer phylogenetic relationship with *M. pasquieri*.

### 3.5. Analysis of Cis-Acting Elements in the MpCesA/Csl Family

**Figure 5:**
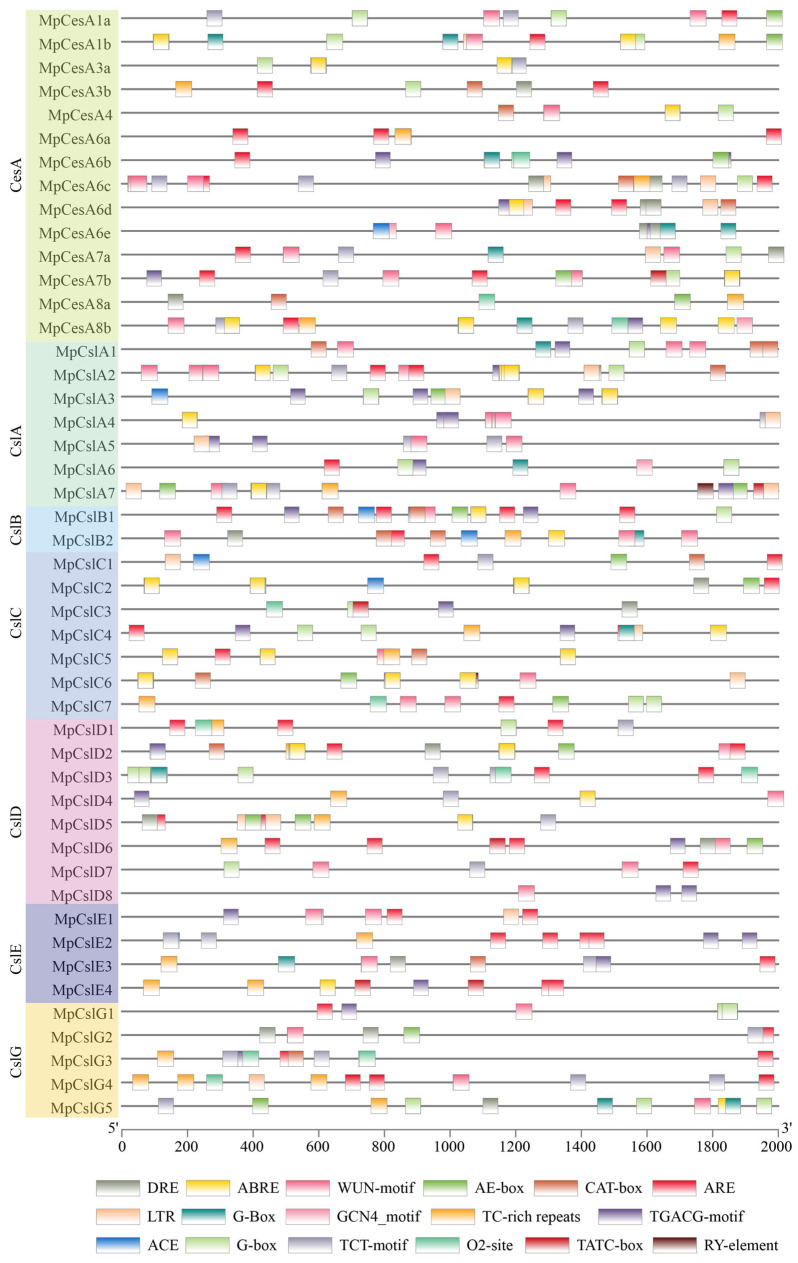
Distribution of cis-acting elements in the promoters of *MpCesA*/*Csl* genes. The gene names are shown on the left, and the annotation information of cis-acting elements is displayed at the bottom.

The 2000 bp upstream sequences of *MpCesA*/*Csl* genes were extracted for cis-acting element analysis, and the distribution of these elements in the upstream regions was visualized (Figure 5). A large number of cis-acting regulatory elements were identified, which are associated with plant hormone responses, light responses, stress responses and plant development. For example, *MpCesA4, MpCesA7a, MpCesA7b* and *MpCesA8b* harbor the ABA-responsive element ABRE, indicating that these genes are likely involved in drought tolerance. Given the canonical function of CesA4/7/8 in secondary cell wall biosynthesis, they may enhance drought resistance by modulating cell wall thickness. Genes such as *MpCesA1b*, *MpCesA3b*, and *MpCesA6b* possess TC-rich repeats in their promoter regions, implying that these genes may participate in defense and stress responses by regulating cell wall remodeling. In addition, light-responsive elements such as AE-box and TCT-motif were abundant among the *MpCesA*/*Csl* gene family. Collectively, this suggests that *MpCesA*/*Csl* genes are involved in the responses of *Madhuca pasquieri* to diverse abiotic and biotic stresses.

### 3.6. Expression of MpCesA/Csl Family in Five Stages of Growth

**Figure 6:**
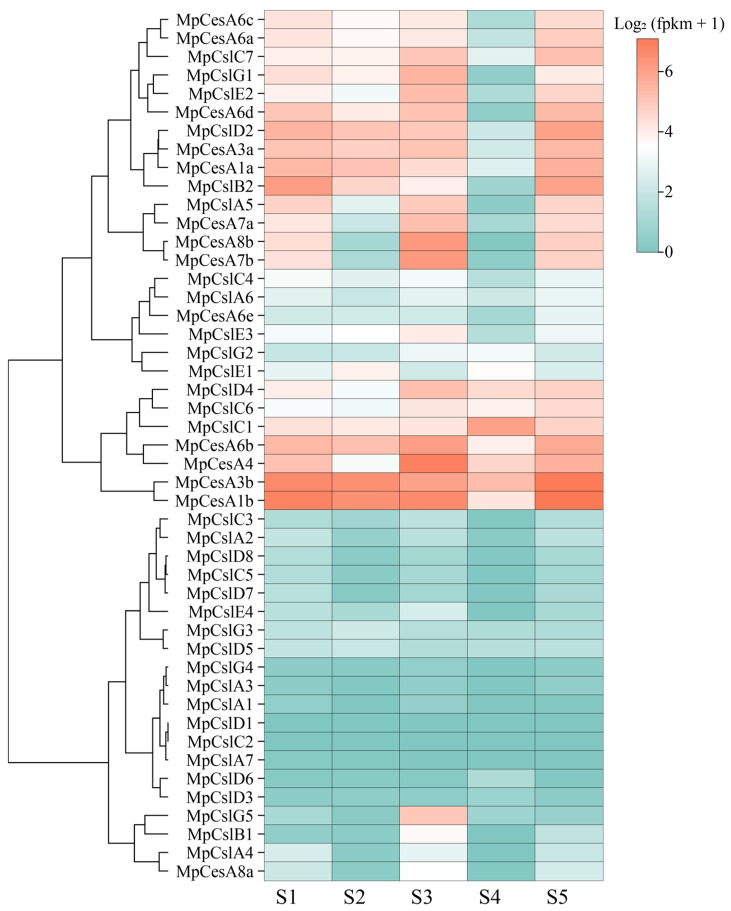
Expression of *MpCesA*/*Csl* Family in five stages. S1: seed germination. S2: hypocotyl elongation. S3: epicotyl elongation. S4: two-leaf stage. S5: nine-leaf stage. Based on the RNA-seq data, gene expression values were visualized in the form of a heatmap, with the color gradient from green to red representing low to high expression levels.

We extracted the transcript expression data of *MpCesA* and *MpCsl* genes based on previously obtained transcriptome datasets covering five successive growth stages of *Madhuca pasquieri* (Table S2). Expression profiling of the *MpCesA*/*Csl* gene family revealed distinct expression patterns across the five samples (Figure 6). A subset of genes, including *MpCesA1b*, *MpCesA3b* and *MpCesA6b*, exhibited constitutively high expression across all samples, suggesting their housekeeping roles in maintaining basic cell wall biosynthesis. In contrast, several genes showed preferential expression in specific samples: for example, *MpCesA7b*/*7a* and *MpCesA8b* were strongly induced in S3, while they were downregulated in S4. In addition, *MpCslA5* showed significant expression levels. Based on its subfamily classification, it is inferred to be involved in the biosynthesis of mannose and glucomannan. *MpCslC1*, *MpCslC6* and *MpCslC7* may participate in the biosynthesis of the required β-1,4-glucan backbone chain. *MpCslD2* and *MpCslD4* may be involved in the synthesis of cellulose or mannose. *MpCslB2* and *MpCslE2* also exhibited notable expression levels. However, due to the limited understanding of their subfamily functions, no further inferences are made here. Notably, a large proportion of genes, particularly those from the *CslA*, *CslD*, and *CslG* subfamilies, displayed low or negligible expression across all samples, implying they may function in specific developmental stages or under specialized conditions not captured in this experiment.

### 3.7. Protein–Protein Interaction Network Analysis of the MpCesA/Csl Family

**Figure 7:**
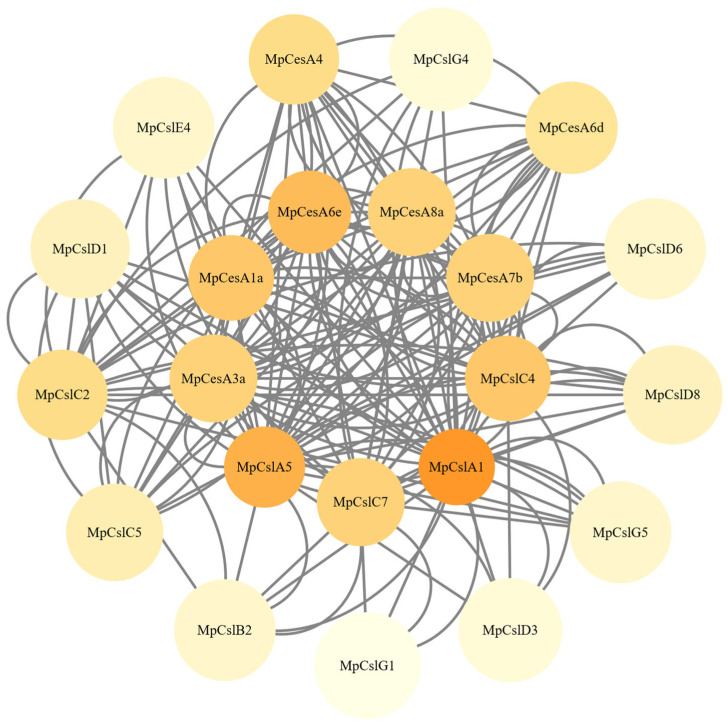
Protein–protein interaction network of the *MpCesA*/*Csl* family. The darker the color and the smaller the circle, the higher the gene connectivity. Genes in the inner circle with higher connectivity are regarded as core nodes.

In this study, protein–protein interaction (PPI) network analysis was performed to systematically characterize the interaction patterns among members of the *CesA*/*Csl* gene family in *M. pasquieri* (Figure 7). *MpCslA1* was identified as the core hub gene of the network, exhibiting a significantly higher degree of connectivity than other family members and serving as a key mediator of interactions among cell wall biosynthesis-related proteins. Core nodes, including *MpCslA4*, *MpCesA7b*, *MpCesA8a*, *MpCslA1* and so on, formed the central module of the network, potentially playing a central regulatory role in the synthesis of cellulose and hemicellulose. Although members of the *CslD*, *CslE*, and *CslG* subfamilies showed relatively low connectivity, they participated in the overall network regulation through interactions with core nodes. These results reveal the synergistic regulatory mechanism of the *CesA*/*Csl* gene family in the cell wall biosynthesis network of *M. pasquieri*.

### 3.8. 3D Structure Analysis of MpCesA/Csl Gene Family Members

**Figure 8:**
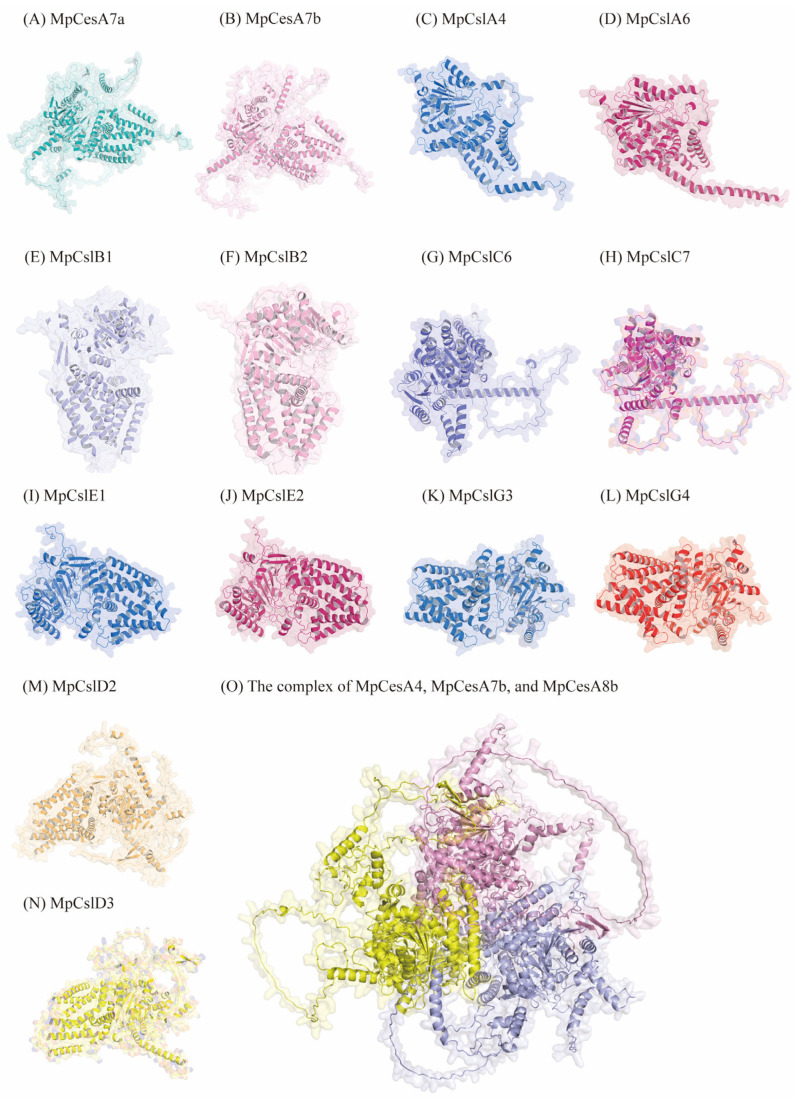
3D structure diagrams of representative proteins in the *MpCesA*/*Csl* family. (**A**) *MpCesA7a*, (**B**) *MpCesA7b*, (**C**) *MpCslA4*, (**D**) *MpCslA6*, (**E**) *MpCslB1*, (**F**) *MpCslB2,* (**G**) *MpCslC6*, (**H**) *MpCslC7*, (**I**) *MpCslE1*, (**J**) *MpCslE2*, (**K**) *MpCslG3*, (**L**) *MpCslG4*, (**M**) *MpCslD2*, (**N**) *MpCslD3*, (**O**) the complex of *MpCesA4*, *MpCesA7b*, and *MpCesA8b*.

Homology modeling was performed using AlphaFold3, and the three-dimensional structures of multiple representative *MpCesA*/*Csl* proteins were successfully obtained (Figure 8). Two genes from each subfamily were selected for in-depth structural analysis, and the results revealed high structural similarity with minimal variation within the same subfamily. Based on transcriptomic data, *MpCesA7b* and *MpCesA8b* had a similar expression pattern, and *CesA* proteins usually perform their function by forming a complex. Therefore, AlphaFold3 was used to resolve the structure of the complex formed by MpCesA4, MpCesA7b, and MpCesA8b, which may be involved in secondary cell wall formation. The analysis yielded an ipTM score of 0.60 and a pTM score of 0.65, indicating high confidence in the interaction among these three proteins (Figure 8O). However, these are only predictions and inferences; whether the complex exists remains questionable.

## 4. Discussion

*M. pasquieri* is a member of the *Madhuca* genus, and relevant studies have been carried out on *M. pasquieri*, *Madhuca longifolia* and *Madhuca hainanensis* [49]. They share several common features and distinct interspecific differences. *M. longifolia* suffers from cold injury [50], whereas the other two species do not, and slow growth is a unique characteristic of *M. pasquieri* that is absent in its two congeners. Although *M. pasquieri* is slow-growing and rare, it has superior wood properties. Plant growth and morphogenesis rely to a certain extent on cell wall expansion, and the *CesA*/*Csl* gene family, as key enzymes, regulates the synthesis rate of cellulose and hemicellulose in plant cell walls. Therefore, genome-wide identification of the *MpCesA*/*Csl* superfamily will not only contribute to the future improvement of wood quality in *M. pasquieri* but also help explore the causes of its slow growth from the perspective of cell wall development. At present, a stable genetic transformation system for *M. pasquieri* has not yet been established, yet our team has made certain progress in tissue culture and is actively exploring feasible transformation systems, which could support the conservation of this endangered tree species through molecular biological strategies.

To construct the phylogenetic tree, *CesA*/*Csl* protein sequences from four species, namely *A. thaliana*, *P. trichocarpa*, *V. vinifera*, and *M. pasquieri*, were used. Among them, *A. thaliana* and *P. trichocarpa* are model plants with well-studied *CesA*/*Csl* families, providing references for gene nomenclature and functional prediction in this study. *V. vinifera*, a liana species, was included to improve the topological stability and analytical reliability of the phylogenetic tree. Phylogenetic analysis showed that the *CesA* subfamily was clearly divided into two distinct clades: the *CesA4*/*7*/*8* clade responsible for secondary cell wall biosynthesis, and the *CesA1*/*3*/*6* clade mainly regulating primary cell wall formation. The potential functions of genes can be inferred from their sequence similarity and phylogenetic relationships in the evolutionary tree. Taking the *CslD* subfamily as an example, previous studies have confirmed that proteins of the *CslD* family participate in cellulose synthesis during root hair and stem growth [51,52,53,54]. Based on phylogenetic clustering, it is speculated that *MpCslD* genes in *M. pasquieri* may also be involved in the growth of root hairs and stems. These enzyme-related genes can be regarded as key candidates for improving the growth rate of the *M. pasquieri* stem. In addition, *AtCslD5* of *A. thaliana* plays a critical role in osmotic stress tolerance by regulating ROS homeostasis under stress conditions [55]. Its homologous gene *OsCslD4* in *O. sativa* enhances osmotic tolerance by mediating abscisic acid (ABA) levels and participates in the salt stress response of rice [56]. Phylogenetic results indicated that *PtCslD1*, *PtCslD2*, *MpCslD7* and *MpCslD8* were clustered together with *AtCslD5*. As woody species, both *P. trichocarpa* and *M. pasquieri* contain two *CslD* copies in this small clade, which is different from *A. thaliana* and *V. vinifera*. These duplicated genes may include non-functional pseudogenes or work together to achieve synergistic effects. Returning to the main topic, it could be inferred that *MpCslD7* and *MpCslD8* may exert vital functions in the osmotic stress response of *M. pasquieri*.

Comparative analysis revealed that the number of *CesA* members varied among species, with 10 in *A. thaliana*, 18 in *P. trichocarpa*, and 14 in *M. pasquieri*. Unlike the herbaceous plant *A. thaliana*, the woody plants *P. trichocarpa* and *M. pasquieri* showed obvious expansion of the *CesA* gene family. Notably, *M. pasquieri* has two duplicated copies of *CesA7* and *CesA8*, named *MpCesA7a*/*7b* and *MpCesA8a*/*8b*, which is highly consistent with *P. trichocarpa*. It is preliminarily inferred that *M. pasquieri* and *P. trichocarpa* may have undergone similar gene duplication events during evolution, leading to the expansion of *CesA* genes, although the underlying molecular mechanism remains to be further explored. Combined with gene duplication characteristics and phylogenetic relationships, we suggest that *M. pasquieri* and *P. trichocarpa* share high conservation in the regulatory mechanism of secondary cell wall formation.

In this study, the expression patterns of *MpCesA*/*Csl* genes were analyzed across five growth periods. A subset of genes, including *MpCesA1b*, *MpCesA3b* and *MpCesA6b*, showed constitutively high expression throughout all stages, suggesting their conserved roles in basic primary cell wall synthesis. In contrast, *MpCesA4*, *MpCesA7b*/*7a*, and *MpCesA8b* were significantly induced during specific stages, indicating their key functions in secondary cell wall thickening. Promoter cis-element analysis revealed that a large number of cis-acting regulatory elements were identified, associated with plant hormone responses, light responses, stress responses and plant development, indicating that the transcription of *MpCesA*/*Csl* genes may be coordinately regulated by developmental and environmental signals.

Transcriptomic analysis in this study has two major limitations. First, the available expression profiles are insufficient, lacking tissue-specific expression patterns and transcriptional data under various treatment conditions. Second, the expression patterns of growth-related genes were not validated by qRT-PCR. Herein, we elaborate on the rationale for the absence of qRT-PCR verification. We have generated single-nucleus transcriptome data from *M. pasquieri* leaves under both control and salicylic acid (SA) treatments and characterized the expression levels of *CesA*/*Csl* genes across distinct cell types. Notably, SA spraying significantly elevated the transcript abundance of *MYB46* and *CesA4*/*7*/*8* in nearly all cell populations. Previous studies have demonstrated that MYB46 acts as a dual-function regulator: it modulates the expression of secondary cellulose synthase genes *CesA4*/*7*/*8* and regulates wax biosynthesis-related genes [57,58]. Therefore, we propose that SA treatment activates *MYB46* as a central regulatory hub, which subsequently upregulates *CesA4*/*7*/*8* in most cell types to promote secondary cell wall thickening. Although the promoter regulation of wax-related genes by *MYB46* has been verified in our work, its transcriptional regulation on *CesA4*/*7*/*8* remains uncharacterized in the present study. Due to the pending publication of our single-nucleus transcriptome dataset, the raw single-cell expression data cannot be displayed here. We still include this evidence to reasonably explain why qRT-PCR validation was not performed. The single-nucleus transcriptomic figures and expression matrix have been submitted as Supplementary Materials and are not presented in the main results, which substantiates the above statements in the Section 4 (Figures S1 and S2, Tables S3 and S4).

Protein–protein interaction (PPI) network analysis showed that *MpCslA1* was the core hub gene with the highest connectivity and, together with *MpCslA4*, *MpCesA7b*, and *MpCesA8a*, formed the central regulatory module. Members of the *CslD*, *CslE*, and *CslG* subfamilies had relatively low connectivity but participated in the overall regulatory network through interactions with core nodes, reflecting synergistic cooperation among family members during cell wall polysaccharide synthesis. Three-dimensional structure modeling based on AlphaFold3 showed that proteins from the same subfamily shared high structural similarity. Importantly, the complex model composed of *MpCesA4*, *MpCesA7b*, and *MpCesA8b* obtained high confidence scores, supporting that these three proteins may form a functional cellulose synthase complex (CSC) on the plasma membrane to catalyze cellulose biosynthesis. Nevertheless, the real structure and assembly mode of CSC need to be further verified by cryo-electron microscopy.

Despite these findings, this study has several limitations. First, all analyses were based on bioinformatics prediction and transcriptome data, lacking in vitro or in vivo functional verification. Second, the PPI network was constructed by homologous mapping to *A. thaliana*, which may not fully reflect the species-specific interaction patterns of *M. pasquieri*. Third, the biological functions of many low-expressed *Csl* genes remain unclear, and their roles in hemicellulose synthesis and stress adaptation need further study. Fourthly, numerous highly homologous gene copies exist in *M. pasquieri*, such as *MpCesA7a*/*7b* and *MpCesA8a*/*8b*. Functional differentiation commonly occurs among these paralogs, and experimental validation is therefore indispensable to identify the exact gene copies that exert essential biological functions.

Future research will focus on functional verification of core hub genes such as *MpCslA1* and secondary wall synthesis genes including *MpCesA4*/*7b*/*7a*/*8a*/*8b* through transgenic or gene-editing methods. We will also conduct supplementary transcriptome sequencing to obtain tissue-specific transcriptomic data and transcriptional profiles under various stress treatments, which can further enrich the gene expression information of *M. pasquieri*. In addition, we will verify the assembly of the cellulose synthase complex (CSC) via biochemical and cytological experiments. These follow-up studies will help systematically clarify the molecular mechanism of cell wall formation and lay a solid foundation for the genetic improvement and resource protection of *M. pasquieri*.

## 5. Conclusions

In this study, based on the high-quality genome of *M. pasquieri*, a total of 47 *MpCesA*/*Csl* genes harboring complete cellulose synthase domains were identified and classified into seven subfamilies, including *CesA*, *CslA*, *CslC*, *CslB*, *CslD*, *CslE*, and *CslG*. Based on the transcriptome data of five growth periods, the expression of *CesA*/*Csl* family was analyzed. Combined with phylogenetic information, it is inferred that *MpCesA4*/*7a*/*7b*/*8b* may regulate the secondary wall, while *MpCesA1*/*3b*/*6b* may regulate the primary wall. Protein–protein interaction showed that *MpCesA4*/*7b*/*8a* were in the core site. Finally, we constructed the cellulose synthase complex (*MpCesA4*/*7b*/*8b*) model using AlphaFold3. It is speculated that *MpCesA4*/*7b*/*8b* may form a complex on the plasma membrane to carry out cellulose synthesis. This study provides a theoretical basis for elucidating the molecular mechanisms by which *CesA*/*Csl* modulate growth and development in *M. pasquieri*, as well as for forest wood quality improvement and biomass utilization.

## Figures and Tables

**Table 1:** Physicochemical Properties and Subcellular Localization Prediction of *MpCesA*/*Csl* Genes.

Name	Sequence ID	Number of Amino Acid	Molecular Weight	Theoretical pI	Instability Index	Aliphatic Index	Grand Average of Hydropathicity
*MpCesA1a*	mikado.chr12G164.1	1071	120,748.5	6.8	43.34	87.01	−0.239
*MpCesA1b*	mikado.chr12G163.1	1072	120,759.5	6.82	42.35	87.2	−0.232
*MpCesA3a*	mikado.chr12G1025.1	1086	121,450.1	7.2	38.14	85.24	−0.194
*MpCesA3b*	mikado.chr1G1910.1	1083	121,159.9	6.99	39.17	84.76	−0.198
*MpCesA4*	mikado.chr12G2243.3	1059	120,014.1	8.02	39.23	80.42	−0.242
*MpCesA6a*	mikado.chr5G1795.1	1097	123,928.7	6.89	38.34	86.77	−0.182
*MpCesA6b*	mikado.chr2G3135.1	1098	123,735.3	6.66	37.24	85.04	−0.206
*MpCesA6c*	mikado.chr6G425.2	1097	123,785.4	6.55	38.83	85.99	−0.21
*MpCesA6d*	mikado.chr6G426.2	1097	123,592.1	6.48	39.68	84.92	−0.207
*MpCesA6e*	mikado.chr2G2445.1	1084	122,111.2	6.54	43.83	86.71	−0.194
*MpCesA7a*	mikado.chr1G3062.2	1090	123,237.6	6.23	42.5	82.17	−0.223
*MpCesA7b*	mikado.chr3G2851.1	1045	118,447.2	6.16	41.75	82.35	−0.212
*MpCesA8a*	mikado.chr4G2750.1	970	109,072.4	5.84	39.5	86.72	−0.112
*MpCesA8b*	mikado.chr4G727.1	982	110,671.3	6.42	37.72	86.15	−0.086
*MpCslA1*	mikado.chr10G2503.1	524	60,182.61	9.1	39.5	94.83	0.114
*MpCslA2*	mikado.chr7G2905.1	526	60,190.03	9.14	38.11	96.88	0.155
*MpCslA3*	mikado.chr1G942.1	534	61,084.68	8.96	34.04	100.92	0.175
*MpCslA4*	mikado.chr9G2209.1	533	60,896.62	9.04	36.98	102.55	0.188
*MpCslA5*	mikado.chr11G990.1	534	61,425.27	9.18	33.43	101.65	0.17
*MpCslA6*	mikado.chr1G941.3	541	62,014.63	7.48	41.64	105.18	0.221
*MpCslA7*	mikado.chr1G837.1	404	46,556.44	9.14	38.41	101.73	0.129
*MpCslB1*	mikado.chr1G4603.2	745	84,155.08	8.12	35.85	86.93	−0.019
*MpCslB2*	mikado.chr1G4606.1	746	84,207.23	8.38	33.88	87.98	−0.005
*MpCslC1*	mikado.chr12G102.1	683	78,603.57	8.89	42.92	100.88	0.054
*MpCslC2*	mikado.chr12G2088.1	691	79,039.69	7.51	46.76	98.89	0.102
*MpCslC3*	mikado.chr2G2095.1	667	76,921.42	8.75	40.53	103.61	0.129
*MpCslC4*	mikado.chr5G3558.1	659	76,255.47	8.95	44.35	100.02	0.081
*MpCslC5*	mikado.chr7G209.4	694	79,077.4	7.54	35.84	103.26	0.12
*MpCslC6*	mikado.chr2G2974.1	701	80,210.26	8.24	43.98	97.22	−0.008
*MpCslC7*	mikado.chr6G1382.1	712	80,915.87	8.34	38.31	95.87	−0.03
*MpCslD1*	mikado.chr5G576.1	1053	118,153.2	8.67	40.32	82.13	−0.243
*MpCslD2*	mikado.chr7G1673.1	1143	128,189.8	6.89	40.03	82.84	−0.219
*MpCslD3*	mikado.chr10G2460.1	1144	128,661.5	6.72	41.56	81.64	−0.222
*MpCslD4*	mikado.chr3G1394.1	1135	126,932.5	7.01	42.28	83.42	−0.18
*MpCslD5*	mikado.chr10G685.4	1139	127,396.9	6.54	41.28	84.07	−0.197
*MpCslD6*	mikado.chr8G1904.1	1138	126,891.4	6.28	41.13	76.61	−0.28
*MpCslD7*	mikado.chr11G2353.1	1173	131,683.6	8.45	44.78	80.47	−0.214
*MpCslD8*	mikado.chr12G2602.1	1171	131,325.2	8.49	42.4	79.94	−0.218
*MpCslE1*	mikado.chr1G4753.1	739	84,122.13	7.95	44.47	89.04	−0.053
*MpCslE2*	mikado.chr4G1639.1	733	83,928.93	7.18	46.12	88.08	−0.092
*MpCslE3*	mikado.chr4G1640.1	734	84,198.2	8.83	39.37	82.33	−0.143
*MpCslE4*	mikado.chr6G1778.1	744	84,558.57	7.47	40.87	89.83	−0.027
*MpCslG1*	mikado.chr3G713.1	735	83,052.04	8.01	38.93	90.07	0.041
*MpCslG2*	mikado.chr9G1225.2	740	83,440.95	8.68	42.09	96.43	0.129
*MpCslG3*	mikado.chr9G1227.1	734	82,872.3	8.64	43.45	97.59	0.129
*MpCslG4*	mikado.chr9G1228.1	733	82,751.3	8.62	41.15	97.74	0.147
*MpCslG5*	mikado.chr5G745.1	712	80,371.46	8.88	40.54	92.47	0.01

**Table 2:** Calculation of Ka/Ks ratios for duplicated gene pairs.

Sequence 1	Sequence 2	Ka	Ks	Ka/Ks	Effective Length
MpCslA6	MpCslA3	0.2548	1.0905	0.2336	1602
MpCslE2	MpCslE3	0.2473	1.0654	0.2321	2175
MpCesA6c	MpCesA6d	0.0093	0.0903	0.1034	3291
MpCslG3	MpCslG4	0.0326	0.0589	0.5534	2199
MpCesA1b	MpCesA1a	0.0057	0.0382	0.1493	3213
MpCesA7a	MpCesA7b	0.0443	0.6710	0.0660	3123
MpCesA8b	MpCesA8a	0.1141	0.5585	0.2043	2883
MpCslC3	MpCslC4	0.0622	0.5500	0.1131	1968
MpCslC6	MpCslC7	0.0641	0.5290	0.1212	2103
MpCslE1	MpCslE4	0.3402	1.5586	0.2183	2199
MpCslD3	MpCslD2	0.0508	0.5458	0.0932	3426
MpCslG5	MpCslG2	0.3886	1.5347	0.2532	2100
MpCslA6	MpCslA4	0.2270	1.0618	0.2137	1572
MpCslA6	MpCslA1	0.2656	1.4787	0.1796	1557
MpCslA3	MpCslA5	0.1689	1.6932	0.0998	1590
MpCesA3b	MpCesA3a	0.0288	0.4389	0.0655	3246
MpCslC1	MpCslC2	0.0900	0.7039	0.1279	2040

## Data Availability

The genome assembly data of *M. pasquieri* generated in this study have been deposited in the Genome Warehouse of the National Genomics Data Center (https://ngdc.cncb.ac.cn), China National Center for Bioinformation. The accession numbers are GWHHIXU00000000.1.

## Supplementary Materials

The following supporting information can be downloaded at: https://www.mdpi.com/article/10.3390/biology15120895/s1. Figure S1. The expression of CesA/Csl in different leaf cells. C1–C24 represent distinct cell clusters. The cell type of each cluster is marked as follows: (A) Mesophyll cell, (B) Epidermal cell, (C) Guard cell, (D) Xylem cell, (E) Phloem cell, (F) Xylem parenchymal cell, (G) Bundle sheath cell, (H) Proliferating cell, (I) Procambium cell, (J) Mesophyll and xylem cell, (K) Mesophyll and bundle sheath cell, (L) Unknown cell. Figure S2. Heatmap of differential *CesA*/*Csl* gene expression in distinct leaf cells under SA treatment. (A) Epidermal Cell, (B) Mesophyll Cell, (C) Guard Cell, (D) Proliferating cell, (E) Phloem cell, (F) Procambium cell, (G) Bundle Sheath cell, (H) Xylem Parenchymal cell, (I) Xylem cell. Table S1. Protein sequences used in this study. Table S2. Expression data of MpCesA/Csl genes. Table S3. Expression data of MpCesA/Csl genes in different clustered cells. Table S4. Expression data of differentially expressed genes across different cell types.

## Abbreviations

The following abbreviations are used in this manuscript:

*CesA*	cellulose synthase
*Csl*	cellulose synthase-like
SA	salicylic acid
CSC	cellulose synthase complex
CDD	Conserved Domain Database
GT2	the Glycosyltransferase 2
PPI	protein–protein interaction
Ka	non-synonymous substitution rate
Ks	synonymous substitution rate
ABA	Abscisic Acid

## References

[1] Cosgrove D.J. (2024). Structure and Growth of Plant Cell Walls. Nat. Rev. Mol. Cell Biol..

[2] Daher F.B., Braybrook S.A. (2015). How to Let Go: Pectin and Plant Cell Adhesion. Front. Plant Sci..

[3] Somerville C., Bauer S., Brininstool G., Facette M., Hamann T., Milne J., Osborne E., Paredez A., Persson S., Raab T. (2004). Toward a Systems Approach to Understanding Plant Cell Walls. Science.

[4] Carroll A., Somerville C. (2009). Cellulosic Biofuels. Annu. Rev. Plant Biol..

[5] Zhong R., Ye Z.-H. (2015). Secondary Cell Walls: Biosynthesis, Patterned Deposition and Transcriptional Regulation. Plant Cell Physiol..

[6] Meents M.J., Watanabe Y., Samuels A.L. (2018). The Cell Biology of Secondary Cell Wall Biosynthesis. Ann. Bot..

[7] Pear J.R., Kawagoe Y., Schreckengost W.E., Delmer D.P., Stalker D.M. (1996). Higher Plants Contain Homologs of the Bacterial celA Genes Encoding the Catalytic Subunit of Cellulose Synthase. Proc. Natl. Acad. Sci. USA.

[8] Zhang S., Jiang Z., Chen J., Han Z., Chi J., Li X., Yu J., Xing C., Song M., Wu J. (2021). The Cellulose Synthase (CesA) Gene Family in Four Gossypium Species: Phylogenetics, Sequence Variation and Gene Expression in Relation to Fiber Quality in Upland Cotton. Mol. Genet. Genom..

[9] Arioli T., Peng L., Betzner A.S., Burn J., Wittke W., Herth W., Camilleri C., Höfte H., Plazinski J., Birch R. (1998). Molecular Analysis of Cellulose Biosynthesis in *Arabidopsis*. Science.

[10] Wang L., Guo K., Li Y., Tu Y., Hu H., Wang B., Cui X., Peng L. (2010). Expression Profiling and Integrative Analysis of the CESA/CSL Superfamily in Rice. BMC Plant Biol..

[11] Xu W., Cheng H., Zhu S., Cheng J., Ji H., Zhang B., Cao S., Wang C., Tong G., Zhen C. (2021). Functional Understanding of Secondary Cell Wall Cellulose Synthases in *Populus trichocarpa* via the Cas9/gRNA-induced Gene Knockouts. New Phytol..

[12] Sod B., Xu L., Liu Y., He F., Xu Y., Li M., Yang T., Gao T., Kang J., Yang Q. (2023). Genome-Wide Identification and Expression Analysis of the CesA/Csl Gene Superfamily in Alfalfa (*Medicago sativa* L.). Agriculture.

[13] De Caroli M., Rampino P., Pecatelli G., Girelli C.R., Fanizzi F.P., Piro G., Lenucci M.S. (2022). Expression of Exogenous GFP-CesA6 in Tobacco Enhances Cell Wall Biosynthesis and Biomass Production. Biology.

[14] Song X., Xu L., Yu J., Tian P., Hu X., Wang Q., Pan Y. (2019). Genome-Wide Characterization of the Cellulose Synthase Gene Superfamily in *Solanum lycopersicum*. Gene.

[15] Hu L., Xu T., Cai Y., Qin Y., Zheng Q., Chen T., Gong L., Yang J., Zhao Y., Chen J. (2025). Identifying Candidate Genes for Grape (*Vitis vinifera* L.) Fruit Firmness through Genome-Wide Association Studies. J. Agric. Food Chem..

[16] An R., Huang Y., Lei C., Wu A.-M., Fan C., Long J. (2025). Genome-Wide Identification and Expression Analysis of the CesA/Csls Gene Family in *Eucalyptus grandis*. Front. Plant Sci..

[17] Pancaldi F., Van Loo E.N., Schranz M.E., Trindade L.M. (2022). Genomic Architecture and Evolution of the Cellulose Synthase Gene Superfamily as Revealed by Phylogenomic Analysis. Front. Plant Sci..

[18] Maleki S.S., Mohammadi K., Movahedi A., Wu F., Ji K.S. (2020). Increase in Cell Wall Thickening and Biomass Production by Overexpression of PmCesA2 in Poplar. Front. Plant Sci..

[19] Taylor N.G., Scheible W.-R., Cutler S., Somerville C.R., Turner S.R. (1999). The *Irregular Xylem3* Locus of Arabidopsis Encodes a Cellulose Synthase Required for Secondary Cell Wall Synthesis. Plant Cell.

[20] Taylor N.G., Laurie S., Turner S.R. (2000). Multiple Cellulose Synthase Catalytic Subunits Are Required for Cellulose Synthesis in Arabidopsis. Plant Cell.

[21] Taylor N.G., Howells R.M., Huttly A.K., Vickers K., Turner S.R. (2003). Interactions among Three Distinct CesA Proteins Essential for Cellulose Synthesis. Proc. Natl. Acad. Sci. USA.

[22] Gardiner J.C., Taylor N.G., Turner S.R. (2003). Control of Cellulose Synthase Complex Localization in Developing Xylem. Plant Cell.

[23] Persson S., Paredez A., Carroll A., Palsdottir H., Doblin M., Poindexter P., Khitrov N., Auer M., Somerville C.R. (2007). Genetic Evidence for Three Unique Components in Primary Cell-Wall Cellulose Synthase Complexes in *Arabidopsis*. Proc. Natl. Acad. Sci. USA.

[24] Kim W., Ko J., Kim J., Kim J., Bae H., Han K. (2013). MYB 46 Directly Regulates the Gene Expression of Secondary Wall-associated Cellulose Synthases in A Rabidopsis. Plant J..

[25] Nixon B.T., Mansouri K., Singh A., Du J., Davis J.K., Lee J.-G., Slabaugh E., Vandavasi V.G., O’Neill H., Roberts E.M. (2016). Comparative Structural and Computational Analysis Supports Eighteen Cellulose Synthases in the Plant Cellulose Synthesis Complex. Sci. Rep..

[26] Gutierrez R., Lindeboom J.J., Paredez A.R., Emons A.M.C., Ehrhardt D.W. (2009). Arabidopsis Cortical Microtubules Position Cellulose Synthase Delivery to the Plasma Membrane and Interact with Cellulose Synthase Trafficking Compartments. Nat. Cell Biol..

[27] Clarke A.E., Anderson M.A., Bacic T., Harris P.J., Mau S.-L. (1985). Molecular Basis of Cell Recognition during Fertilization in Higher Plants. J. Cell Sci..

[28] Yin Y., Johns M.A., Cao H., Rupani M. (2014). A Survey of Plant and Algal Genomes and Transcriptomes Reveals New Insights into the Evolution and Function of the Cellulose Synthase Superfamily. BMC Genom..

[29] Little A., Schwerdt J.G., Shirley N.J., Khor S.F., Neumann K., O’Donovan L.A., Lahnstein J., Collins H.M., Henderson M., Fincher G.B. (2018). Revised Phylogeny of the *Cellulose Synthase* Gene Superfamily: Insights into Cell Wall Evolution. Plant Physiol..

[30] Liepman A.H., Wilkerson C.G., Keegstra K. (2005). Expression of Cellulose Synthase-like (*Csl*) Genes in Insect Cells Reveals That *CslA* Family Members Encode Mannan Synthases. Proc. Natl. Acad. Sci. USA.

[31] Goubet F., Barton C.J., Mortimer J.C., Yu X., Zhang Z., Miles G.P., Richens J., Liepman A.H., Seffen K., Dupree P. (2009). Cell Wall Glucomannan in Arabidopsis Is Synthesised by CSLA Glycosyltransferases, and Influences the Progression of Embryogenesis. Plant J..

[32] Cocuron J.-C., Lerouxel O., Drakakaki G., Alonso A.P., Liepman A.H., Keegstra K., Raikhel N., Wilkerson C.G. (2007). A Gene from the Cellulose Synthase-like C Family Encodes a β-1,4 Glucan Synthase. Proc. Natl. Acad. Sci. USA.

[33] Yang J., Bak G., Burgin T., Barnes W.J., Mayes H.B., Peña M.J., Urbanowicz B.R., Nielsen E. (2020). Biochemical and Genetic Analysis Identify CSLD3 as a Beta-1,4-Glucan Synthase That Functions during Plant Cell Wall Synthesis. Plant Cell.

[34] Little A., Lahnstein J., Jeffery D.W., Khor S.F., Schwerdt J.G., Shirley N.J., Hooi M., Xing X., Burton R.A., Bulone V. (2019). A Novel (1,4)-β-Linked Glucoxylan Is Synthesized by Members of the *Cellulose Synthase-like F* Gene Family in Land Plants. ACS Cent. Sci..

[35] Lou H., Tucker M.R., Shirley N.J., Lahnstein J., Yang X., Ma C., Schwerdt J., Fusi R., Burton R.A., Band L.R. (2022). The *Cellulose Synthase-like F3* (*CslF3*) Gene Mediates Cell Wall Polysaccharide Synthesis and Affects Root Growth and Differentiation in Barley. Plant J..

[36] Richmond T.A., Somerville C.R. (2000). The Cellulose Synthase Superfamily. Plant Physiol..

[37] Kan L., Liao Q., Su Z., Tan Y., Wang S., Zhang L. (2020). Single-Molecule Real-Time Sequencing of the *Madhuca pasquieri* (Dubard) Lam. Transcriptome Reveals the Diversity of Full-Length Transcripts. Forests.

[38] Kan L., Liao Q., Chen Z., Wang S., Ma Y., Su Z., Zhang L. (2021). Dynamic Transcriptomic and Metabolomic Analyses *of Madhuca pasquieri* (Dubard) H. J. Lam During the Post-Germination Stages. Front. Plant Sci..

[39] Chen C., Wu Y., Li J., Wang X., Zeng Z., Xu J., Liu Y., Feng J., Chen H., He Y. (2023). TBtools-II: A “One for All, All for One” Bioinformatics Platform for Biological Big-Data Mining. Mol. Plant.

[40] Duvaud S., Gabella C., Lisacek F., Stockinger H., Ioannidis V., Durinx C. (2021). Expasy, the Swiss Bioinformatics Resource Portal, as Designed by Its Users. Nucleic Acids Res..

[41] Horton P., Park K.-J., Obayashi T., Fujita N., Harada H., Adams-Collier C.J., Nakai K. (2007). WoLF PSORT: Protein Localization Predictor. Nucleic Acids Res..

[42] Price M.N., Dehal P.S., Arkin A.P. (2009). FastTree: Computing Large Minimum Evolution Trees with Profiles Instead of a Distance Matrix. Mol. Biol. Evol..

[43] Xie J., Chen Y., Cai G., Cai R., Hu Z., Wang H. (2023). Tree Visualization By One Table (tvBOT): A Web Application for Visualizing, Modifying and Annotating Phylogenetic Trees. Nucleic Acids Res..

[44] Bailey T.L., Johnson J., Grant C.E., Noble W.S. (2015). The MEME Suite. Nucleic Acids Res..

[45] Lescot M. (2002). PlantCARE, a Database of Plant Cis-Acting Regulatory Elements and a Portal to Tools for in Silico Analysis of Promoter Sequences. Nucleic Acids Res..

[46] Szklarczyk D., Kirsch R., Koutrouli M., Nastou K., Mehryary F., Hachilif R., Gable A.L., Fang T., Doncheva N.T., Pyysalo S. (2023). The STRING Database in 2023: Protein–Protein Association Networks and Functional Enrichment Analyses for Any Sequenced Genome of Interest. Nucleic Acids Res..

[47] Shannon P., Markiel A., Ozier O., Baliga N.S., Wang J.T., Ramage D., Amin N., Schwikowski B., Ideker T. (2003). Cytoscape: A Software Environment for Integrated Models of Biomolecular Interaction Networks. Genome Res..

[48] Abramson J., Adler J., Dunger J., Evans R., Green T., Pritzel A., Ronneberger O., Willmore L., Ballard A.J., Bambrick J. (2024). Accurate Structure Prediction of Biomolecular Interactions with AlphaFold 3. Nature.

[49] Ma Y., Wang Z., Qiu J., Ye S., Chen G., Qin J., Zhang L. (2026). Chromosome-Level Genome of Madhuca Hainanensis Reveals Genomic Evolution and Floral Divergence in Madhuca. iScience.

[50] Chen Y., Qin J., Wang Z., Lin H., Ye S., Wei J., Wang S., Zhang L. (2025). Genome-Wide Identification of 109 NAC Genes and Dynamic Expression Profiles Under Cold Stress in *Madhuca longifolia*. Int. J. Mol. Sci..

[51] Favery B., Ryan E., Foreman J., Linstead P., Boudonck K., Steer M., Shaw P., Dolan L. (2001). *KOJAK* Encodes a Cellulose Synthase-like Protein Required for Root Hair Cell Morphogenesis in *Arabidopsis*. Genes Dev..

[52] Wang X., Cnops G., Vanderhaeghen R., De Block S., Van Montagu M., Van Lijsebettens M. (2001). *AtCSLD3*, A Cellulose Synthase-Like Gene Important for Root Hair Growth in Arabidopsis. Plant Physiol..

[53] Bernal A.J., Jensen J.K., Harholt J., Sørensen S., Moller I., Blaukopf C., Johansen B., De Lotto R., Pauly M., Scheller H.V. (2007). Disruption of *ATCSLD5* Results in Reduced Growth, Reduced Xylan and Homogalacturonan Synthase Activity and Altered Xylan Occurrence in Arabidopsis. Plant J..

[54] Kim C.M., Park S.H., Je B.I., Park S.H., Park S.J., Piao H.L., Eun M.Y., Dolan L., Han C. (2007). *OsCSLD1*, a Cellulose Synthase-Like D1 Gene, Is Required for Root Hair Morphogenesis in Rice. Plant Physiol..

[55] Zhu J., Lee B.-H., Dellinger M., Cui X., Zhang C., Wu S., Nothnagel E.A., Zhu J.-K. (2010). A Cellulose Synthase-like Protein Is Required for Osmotic Stress Tolerance in Arabidopsis: SOS6 Is Important for Osmotic Stress Tolerance in Plants. Plant J..

[56] Zhao H., Li Z., Wang Y., Wang J., Xiao M., Liu H., Quan R., Zhang H., Huang R., Zhu L. (2022). Cellulose Synthase-like Protein OsCSLD4 Plays an Important Role in the Response of Rice to Salt Stress by Mediating Abscisic Acid Biosynthesis to Regulate Osmotic Stress Tolerance. Plant Biotechnol. J..

[57] Jin S., Wang Y., Song Y., Fan S., Luo N., Gan Q., Fan Y., Guo Y., Ni Y. (2025). Dual Regulation of Cuticle and Cell Wall Biosynthesis by BnaC9.MYB46 Confers Drought Tolerance in *Brassica napus*. Plant Biotechnol. J..

[58] Jin S., Song Y., Wang Y., Guo Y., Fan S., Gan Q., Luo N., Fan Y., Ni Y. (2025). MYB46 Integrates Cuticle and Cell Wall Remodeling to Coordinate Drought Tolerance and Pathogen Resistance in *Arabidopsis*. New Phytol..

