# Supplementary material for: Genome-Wide Identification and Expression Analysis of the CesA/Csl Superfamily in Madhuca pasquieri
Source: Biology (Basel). 2026 Jun 6;15(12):895. doi: 10.3390/biology15120895 (PMC13295593; doi:10.3390/biology15120895)
Supplement: Supplementary file 1 [file biology-15-00895-s001.zip › Figure S1 and S2.pdf]

Expression patterns of *MpCesA*/*Csl* in various leaf cells

Based on our in-house leaf single-nucleus transcriptome data, we characterized the expression profiles of the *MpCesA/Csl* family genes in different cell clusters and annotated the correspondence between cell clusters and cell types (Table S3). Genes display divergent expression patterns across distinct clusters (Figure S1). Closely related genes or duplicated gene pairs, such as *MpCesA7a/7b* and *MpCslA1/A6*, exhibited similar expression characteristics. In particular, *MpCesA4*, *MpCesA7b*, *MpCesA8b* and *MpCesA7a* were highly expressed in leaf xylem cells, suggesting that these genes may collaboratively participate in secondary cell wall biosynthesis.

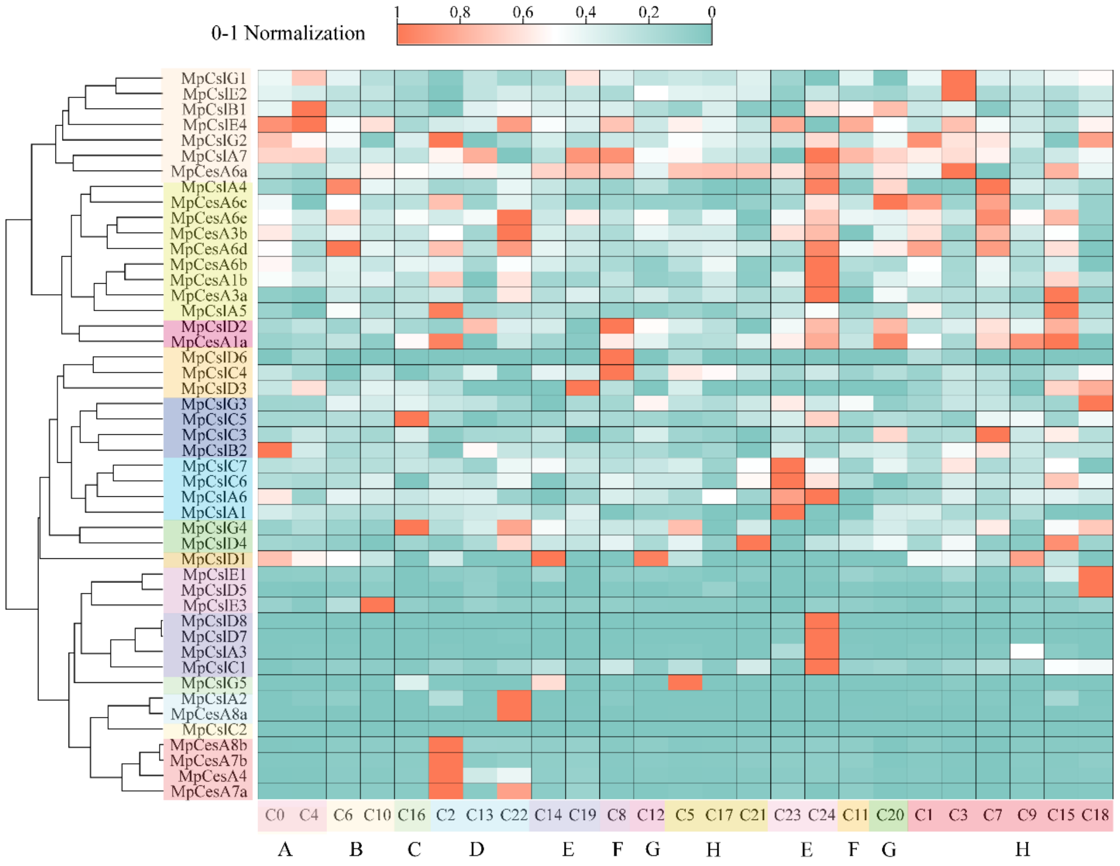

Figure S1. The expression of CesA/Csl in different leaf cells. C1–C24 represent distinct cell clusters. The cell type of each cluster is marked as follows: (A) Mesophyll cell, (B) Epidermal cell, (C) Guard cell, (D) Xylem cell, (E) Phloem cell, (F) Xylem parenchymal cell, (G) Bundle sheath cell, (H) Proliferating cell, (I) Procambium cell, (J) Mesophyll and xylem cell, (K) Mesophyll and bundle sheath cell, (L) Unknown cell.

Expression changes of *MpCesA/Csl* in distinct leaf cells upon SA treatment

To explore the response of the *MpCesA/Csls* gene family to phytohormone treatment, leaves of *Madhuca pasquieri* were sprayed with salicylic acid (SA), and

samples were collected at 24 h after treatment. Single-nucleus transcriptome sequencing was performed on leaf samples from the control and SA-treated groups (Table S4). We generated heatmaps of differentially expressed genes in various cell types (Figure S2). The results showed that most *CesA/Csl* genes were significantly upregulated in the majority of cell types, while only a small number of genes were downregulated in a few specific cells. The types and numbers of hormone-responsive genes varied among different cells, implying that leaf cells form a complex regulatory network to cope with phytohormone stimulation. Notably, *MpCesA4*, *MpCesA7b*, *MpCesA8b*, and *MpMYB46* were dramatically induced in nearly all cell types. We hypothesized that MpMYB46-mediated regulation may activate the expression of *MpCesA4*, *MpCesA7b* and *MpCesA8b*, thereby promoting secondary cell wall thickening.

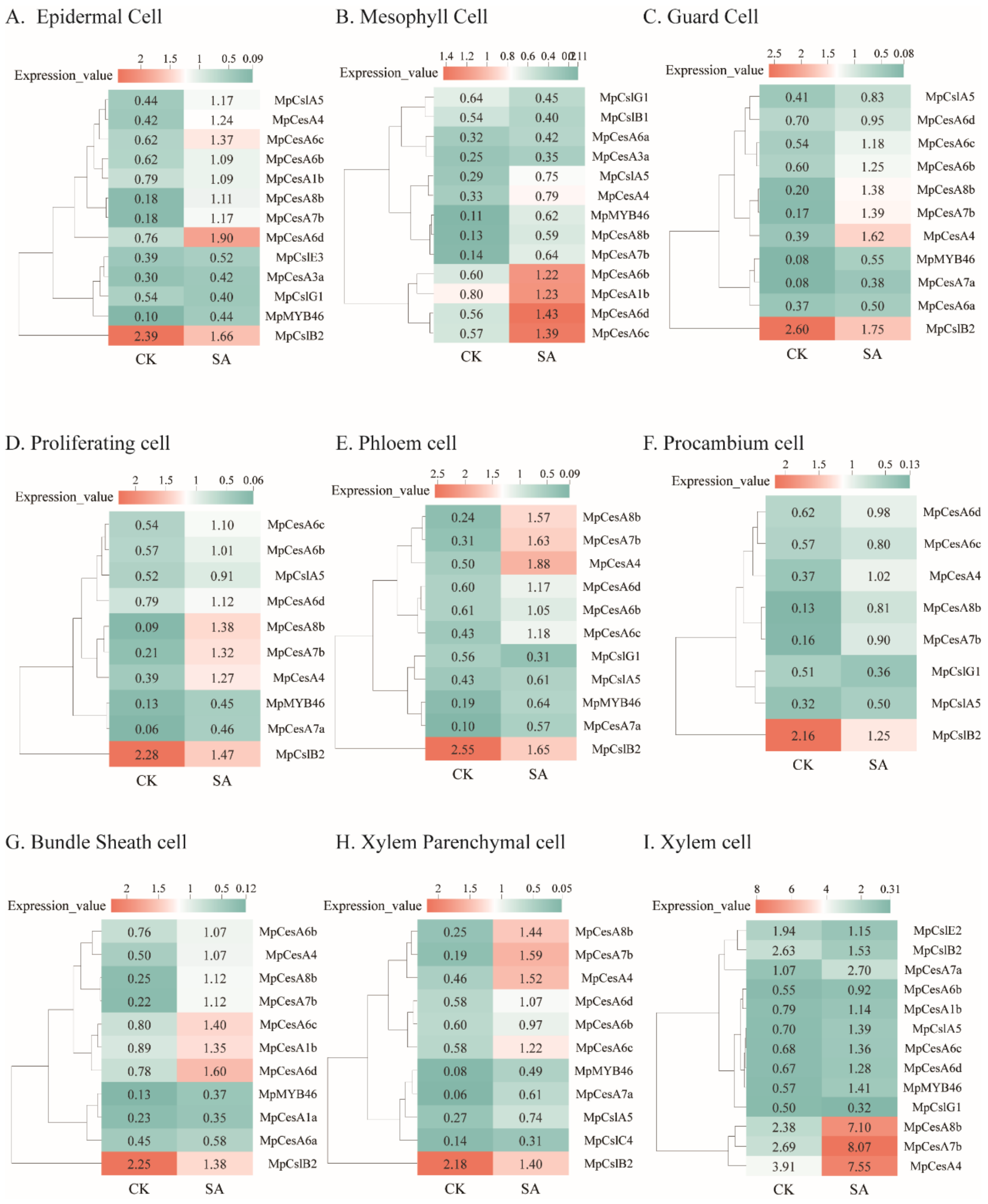

Figure S2. Heatmap of differential *CesA*/*Csl* gene expression in distinct leaf cells under SA treatment. (A) Epidermal Cell, (B) Mesophyll Cell, (C) Guard Cell, (D) Proliferating cell, (E) Phloem cell, (F) Procambium cell, (G) Bundle Sheath cell, (H) Xylem Parenchymal cell, (I) Xylem cell.
